# One-Pot Solvothermal Synthesis of Highly Emissive, Sodium-Codoped, LaF_3_ and BaLaF_5_ Core-Shell Upconverting Nanocrystals

**DOI:** 10.3390/nano4010069

**Published:** 2014-01-08

**Authors:** Joshua T. Stecher, Anne B. Rohlfing, Michael J. Therien

**Affiliations:** Department of Chemistry, Duke University, French Family Science Center, 124 Science Drive, Durham, NC 27708, USA; E-Mails: joshua.stecher@duke.edu (J.T.S.); anne.rohlfing@duke.edu (A.B.R.)

**Keywords:** near-infrared to ultraviolet upconversion, photoluminescence, upconversion nanocrystals, impurity doping, lanthanide, LaF_3_, BaLaF_5_, core-shell

## Abstract

We report a one-pot solvothermal synthesis of sub-10 nm, dominant ultraviolet (UV) emissive upconverting nanocrystals (UCNCs), based on sodium-codoped LaF_3_ and BaLaF_5_ (0.5%Tm; 20%Yb) and their corresponding core@shell derivatives. Elemental analysis shows a Na-codopant in these crystal systems of ~20% the total cation content; X-ray diffraction (XRD) data indicate a shift in unit cell dimensions consistent with these small codopant ions. Similarly, X-ray photoelectron spectroscopic (XPS) analysis reveals primarily substitution of Na^+^ for La^3+^ ions (97% of total Na^+^ codopant) in the crystal system, and interstitial Na^+^ (3% of detected Na^+^) and La^3+^ (3% of detected La^3+^) present in (Na)LaF_3_ and only direct substitution of Na^+^ for Ba^2+^ in Ba(Na)LaF_5_. In each case, XPS analysis of La 3d lines show a decrease in binding energy (0.08–0.25 eV) indicating a reduction in local crystal field symmetry surrounding rare earth (R.E.^3+^) ions, permitting otherwise disallowed R.E. UC transitions to be enhanced. Studies that examine the impact of laser excitation power upon luminescence intensity were conducted over 2.5–100 W/cm^2^ range to elucidate UC mechanisms that populate dominant UV emitting states. Low power saturation of Tm^3+^ ^3^F_3_ and ^3^H_4_ states was observed and noted as a key initial condition for effective population of the ^1^D_2_ and ^1^I_6_ UV emitting states, via Tm-Tm cross-relaxation.

## 1. Introduction

The design of upconverting nanocrystals (UCNCs) with specific, tunable electronic spectroscopic profiles [1,2,3,4], based on multiple dopants and mixed host crystal structures [5,6], has shown great promise for applications that range from biomedical detection [7,8,9] to specifically triggered photoresponsive reactions [10,11]. The advantages of rare earth-based UCNCs capable of near-infrared (NIR) sensitization [excitation wavelength (λ_ex_) = 980 nm] of a ytterbium dopant have been well documented in the literature, and are exemplified by the capability of activating alternate emitting rare earth (R.E.) ions (e.g., Ho, Er, Tm) through 4f-4f phonon-assisted energy transfer processes; these non-resonant spin and Laporte forbidden processes result in anti-Stokes emission from multiple excited R.E. f-block states [3,12,13]. Due to the long-lived excited states of R.E. dopants (μs–ms), multiple R.E. emitting ions are capable of undergoing complex upconversion and relaxation processes in concert [13,14,15], producing a range of emission profiles via energy transfer reactions that give rise to high energy R.E.-based emissive states [16,17], and through activation of tandem organic chromophores [18].

As R.E.-based upconversion relies on atomic 4f-orbital emitting states, emission wavelengths are essentially fixed; modulation of UCNC composition provides control of both the relative intensity of emissive states and the nature of the multi-colored emission over the UV-vis-NIR spectral regime [1,2,3,4]. However, as energy transfer upconversion (ETU) processes require the assistance of host crystal phonon modes to aid in energetically matching non-resonant transitions, tuning of f-block transitions on the order of several hundred wavenumbers can be achieved with varied crystal systems. Recent work has shown general enhancement of upconversion luminescence through doping of relatively small, non-emitting ions (Li, Sc, Ca, *etc*.) in low concentrations, which serve to either replace ions in the crystal lattice or occupy interstitial sites. Interstitial site occupation by these dopants leads to lattice distortions and a reduction in crystal field symmetry [19,20,21,22], as evidenced by X-ray diffraction (XRD) and X-ray photoelectron spectroscopic (XPS) studies. Diminished symmetry proximal to emitting R.E. ions permits otherwise disallowed ETU transitions to become more favorable. These studies have reported between two- and 50-fold enhancements of luminescence intensity in two-photon upconversion processes extending from the NIR to visible spectral regime [19,20,21,22], but do not address the potential for enhancement of higher energy, UV emitting transitions or their possible upconversion mechanisms.

While excellent progress has been made regarding surface modifications of non-toxic UCNCs [23,24,25] for biological applications [26,27], conjugation of UCNCs to organic chromophores [18,28] and photoactive drug moieties [10,18,23], enhancement of specific high energy, UV R.E. ion emission bands has proven challenging. Though dominant UV emission has been achieved in Tm/Yb codoped upconverter compositions in 80 nm, 12 µm, and bulk [29,30,31] size regimes, these materials capitalize on low surface area to volume ratios and complementary diminished surface quenching characteristic of UCNCs having diameters greater than 50 nm [32,33]. Due to the reliance on small surface area:volume ratios, it has proven challenging to generate small, dominant UV emitting UCNCs; to date, there are no known examples in the literature of sub-10 nm UCNCs with a dominant UV emission profile. To this end, we introduce sub-10 nm UCNCs of sodium-codoped LaF_3_ (0.5%Tm, 20%Yb) and BaLaF_5_ (0.5%Tm, 20%Yb) and their core@shell derivatives, generated via a one-pot solvothermal approach, which exhibit dominant UV emission or broadly tunable UV-vis-NIR emission profiles. High sodium codopant (>20% *versus* rare earth content) upconverting materials are shown to enhance UV emission intensity from ^1^I_6_ → ^3^F_4_ (349 nm) and ^1^D_2_ → ^3^H_6_ (362 nm) Tm^3+^ transitions by 800- and 540-fold in (Na)LaF_3_ and 65- and 50-fold in Ba(Na)LaF_5_, respectively, compared to their non-sodium included analogues. Additionally, studies of laser excitation power density *vs*. luminescence intensity, plotted on a ln-ln scale, provide insight into the number of ETU events required to populate known Tm^3+^ emissive states. Similar power dependence studies of upconverted emission intensity conducted on the core@shell materials *vs*. their unshelled counterparts reveal shifts in the upconversion mechanism at low laser densities (<30 W/cm^2^), demonstrating improved efficiency of upconversion into high energy states, as evinced by a decreased number of Yb-to-Tm ETU processes required to populate these states, as well as relaxation processes that produce comparatively strong NIR emissions. Both of these properties highlight the suitability of these nanomaterials for applications requiring dominant UV emission with concomitant intense NIR emission.

## 2. Results and Discussion

### 2.1. UCNC Materials and Emission Profiles

**Figure 1:**
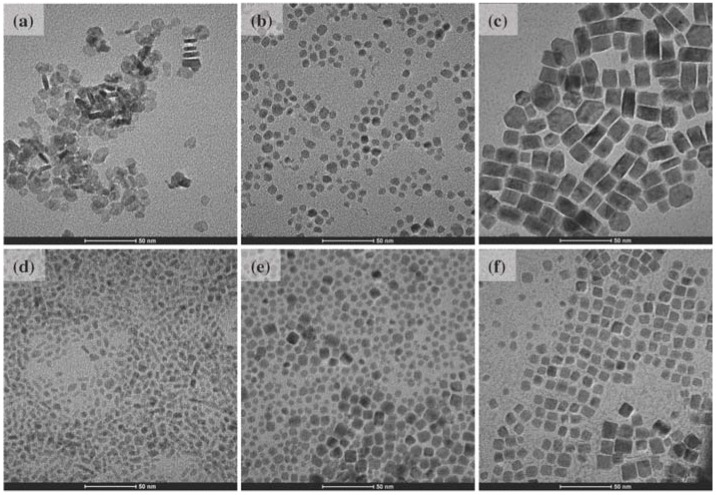
Transmission electron microscopy (TEM) images of 0.5%Tm, 20%Yb codoped Upconverting Nanocrystals (UCNCs) in host lattices: (**a**) LaF_3_ (12.6 ± 2.5 nm); (**b**) (Na)LaF_3_ (7.7 ± 1.9 nm); (**c**) (Na)LaF_3_@(Na)LaF_3_ (22.7 ± 4.5 nm); (**d**) BaLaF_5_ (6.0 ± 1.4 nm); (**e**) Ba(Na)LaF_5_ (7.5 ± 2.3 nm); (**f**) Ba(Na)LaF_5_@Ba(Na)LaF_3_ (9.6 ± 2.3 nm): (200 kx magnification; 50 nm scale bars).

**Figure 2:**
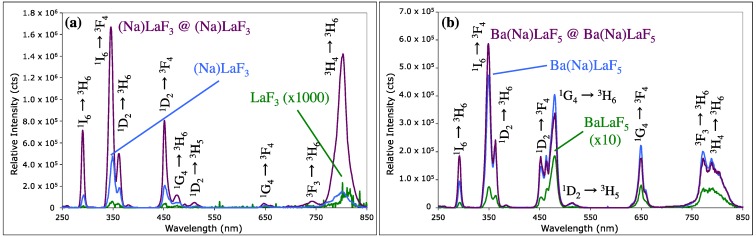
Comparative emission spectra of 0.5%Tm, 20%Yb codoped UCNCs in host lattices. (**a1**) LaF_3_ (12.6 ± 2.5 nm) (intensity shown scaled ×1000) (**Green**); (**a2**) (Na)LaF_3_ (7.7 ± 1.9 nm) (**Blue**); (**a3**) (Na)LaF_3_@(Na)LaF_3_ (22.7 ± 4.5 nm) (**Violet**); (**b1**) BaLaF_5_ (6.0 ± 1.4 nm) (intensity shown scaled ×10 (**Green**); (**b2**) Ba(Na)LaF_5_ (7.5 ± 2.3 nm) (**Blue**); (**b3**) Ba(Na)LaF_5_@Ba(Na)LaF_3_ (9.6 ± 2.3 nm) (**Violet**); experimental conditions: 1 mg/mL solutions in toluene; 980 nm CW laser excitation; 60 W/cm^2^. Tm^3+^ transitions assigned.

Sodium codoped LaF_3_ and BaLaF_5_ (herein denoted as (Na)LaF_3_ and Ba(Na)LaF_5_) UCNCs were generated via a solvothermal synthesis, similar to that reported by Wang *et al*. (see Section 3.1) [34]. Both (Na)LaF_3_ and Ba(Na)LaF_5_ materials were doped with 0.5%Tm and 20%Yb, concentrations, *versus* total R.E., optimized to produce the highest emission intensity from the ^1^I_6_ → ^3^F_4_ (349 nm) and ^1^D_2_ → ^3^H_6_ (362 nm) Tm^3+^ transitions. LaF_3_ and BaLaF_5_ UCNCs, synthesized in the absence of sodium in the same size regime, were similarly doped with 0.5%Tm and 20%Yb (Figure 1) to minimize any possible surface defect site variability as a cause of luminescence quenching (Figure S1). Shells of (Na)LaF_3_ and Ba(Na)LaF_5_ host crystals were solvothermally grown following initial preparation of core seed crystals, in the presence of excess fluoride, in the same Teflon^®^-lined autoclavable bombs through addition of aqueous La(NO_3_)_3_ and Ba(NO_3_)_2_ salts with no intermediate purification required (see Section 3.1). These shells were applied, congruent with earlier studies, to reduce the number of surface defect trap sites capable of quenching surface presenting R.E. ion emission or quenching of core-residing R.E. excited states through R.E.-to-R.E. energy migration to the surface, thereby increasing the overall luminescence output (Figure 2) [8,18,35,36,37].

In each case, compared to 0.5%Tm, 20%Yb doped nanocrystals lacking a sodium codopant, these materials demonstrate significant luminescence enhancement at identical 60 W/cm^2^, 980 nm CW laser excitation conditions. As sodium codopant is introduced into the host crystal lattice, a noted shift in relative emission intensity is seen from lower energy Tm^3+^ transitions ^3^H_4_ → ^3^H_6_ (801 nm) in LaF_3_ and ^1^G_4_ → ^3^H_6_ (480 nm) in BaLaF_5_ to the ^1^I_6_ → ^3^F_4_ (349 nm) transition (Figure 2).

Global emission enhancement of two orders of magnitude is observed in the case of (Na)LaF_3_ compared to LaF_3_ (Figure 2a, Blue and Green traces, respectively). More noteworthy is the appearance of dominant UV emission (349 nm; ^1^I_6_ → ^3^F_4_) over that of the NIR (776–801 nm; ^3^F_3_ → ^3^H_6_ and ^3^H_4_ → ^3^H_6_ transitions), and an enhancement of 800-fold in the ^1^I_6_ → ^3^F_4_ emissive transition in the sodium-codoped LaF_3_ over that characteristic of the control. Even under modest 20 W/cm^2^ 980 nm excitation conditions, the ^1^I_6_ → ^3^F_4_, 349 nm transition displays greater intensity than the NIR emissive transitions derived from two-photon ETU processes. The UV manifold of Na-codoped *versus* control BaLaF_5_ (highlighted in Figure 2b) shows a relative emission enhancement of 65-fold in the ^1^I_6_ → ^3^F_4_ transition, with a similar observed dominant UV emissive signature.

As precedent literature has shown, further enhancement of luminescence can be achieved by shelling UCNCs with a solvothermally grown host crystal, lacking in R.E. emitting ions [8,15,35,37,38,39]. This shelling both diminishes the extent of vibrational relaxation processes involving electronically excited, R.E. dopants and solvent, and augments the emitting ion separation distance from surface crystal defect sites, which can diminish the extent of defect-site luminescence quenching and potentially impact the nature of phonon-assisted energy transfer through the host crystal. Applying a thin shell of inorganic host crystal augments dominant UV emission with nanocrystal sizes below 30 nm, avoiding the need to generate 100 nm-to-µm sized UCs that feature greater concentrations of emitting ions within the UCNC core relative to the surface, where non-radiative relaxation processes dominate. Oftentimes the enhancement of UC emission through inorganic shelling favors, proportionally, the more dominant emissive processes, as is observed for Ba(Na)LaF_5_@Ba(Na)LaF_5_. In the case of (Na)LaF_3_@(Na)LaF_3_, however, a disproportionate enhancement of the NIR ^3^F_3_ → ^3^H_6_ and ^3^H_4_ → ^3^H_6_ transitions (9.6-fold) over the dominant ^1^I_6_ → ^3^F_4_, UV transition (2.7-fold) is seen.

The UC emission enhancements observed through the introduction of sodium-codopant into the host crystals and solvothermal shelling of UCNC cores derives from shifts in the relative populations of emitting states. The distribution of excited state populations can be affected via several factors, including shifts in unit cell dimensions and crystallographic phases, alteration of crystal field symmetry surrounding the emitting Tm^3+^ ions, and modulation of the excited state lifetimes of either the Yb^3+^ sensitizer or Tm^3+^ activator caused by changes to the local crystal field environment.

### 2.2. Crystal Structure and Emissive Ion Environment

**Figure 3:**
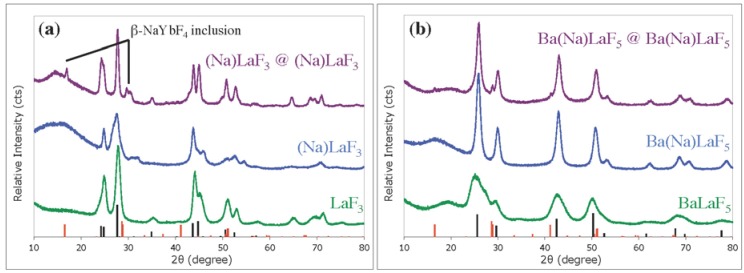
X-ray powder diffraction (XRD) data acquired for: (**a1**) LaF_3_ (**Green**); (**a2**) (Na)LaF_3_ (**Blue**); (**a3**) (Na)LaF_3_@(Na)LaF_3_ (**Violet**); (**a4**) line spectrum of hexagonal LaF_3_, JCPDS: 72-1435 (**Black**); hexagonal NaYbF_4_ inclusions denoted in (Na)LaF_3_@(Na)LaF_3_: (**b1**) BaLaF_5_ (**Green**); (**b2**) Ba(Na)LaF_5_ (**Blue**); (**b3**) Ba(Na)LaF_5_@Ba(Na)LaF_3_ (**Violet**); (**b4**) displays JCPDS: 43-0394 (cubic BaCeF_5_ comparative, **Black**); and (**a5**,**b5**) line spectrum of hexagonal NaLaF_4_, JCPDS: 75-1923 (**Red**).

Unlike previous reports of Ca^2+^ and Ba^2+^ codoping of R.E.F_3_, wherein the crystal structure is changed from hexagonal to cubic and a resultant shift in both unit cell dimensions and crystal field environment is seen [21], Na-codoping into (Na)LaF_3_ does not drive a compositional or phase change to NaLaF_4_, nor does Ba(Na)LaF_5_ show hexagonal NaLaF_4_ impurities (Figure 3). Most notably, Na-codoping of LaF_3_ results in a decrease in 2θ values of all crystallographic peaks (Figure S2) compared to the undoped control, indicating an increase in unit cell dimension most likely due to Na^+^ insertion into interstitial sites in the hexagonal unit cell, though direct La^3+^ to Na^+^ substitution is also expected [19,22,40,41]. The reverse trend can be seen in Ba(Na)LaF_5_, where an increase in 2θ values (Table S1) indicates a reduction in unit cell volume, likely through substitution of Ba^2+^ ions for Na^+^ [19,20,42,43]. Inductively coupled plasma optical emission spectroscopy (ICP-OES) elemental analysis of (Na)LaF_3_ and Ba(Na)LaF_5_ (Table 1) indicates a high level of Na-codopant (>20%) in both UCNC systems, and a marked replacement of Ba^2+^ ions for Na^+^ in the Ba(Na)LaF_5_ UCNC cores. In contrast to the expected 1:1 ratio of Ba:rare earth (R.E.) elements (La, Tm, and Yb) in BaLaF_5_, a 0.74:1 (Ba:R.E.) ratio was determined, along with a 0.60:1 Na:R.E. molar ratio. Either by substitution of La^3+^ or Ba^2+^, insertion into interstitial sites, or a combination of the two, the change in unit cell dimensions would decrease the crystal field symmetry surrounding emitting Tm^3+^ ions and facilitate luminescence enhancement [19,40].

**Figure 4:**
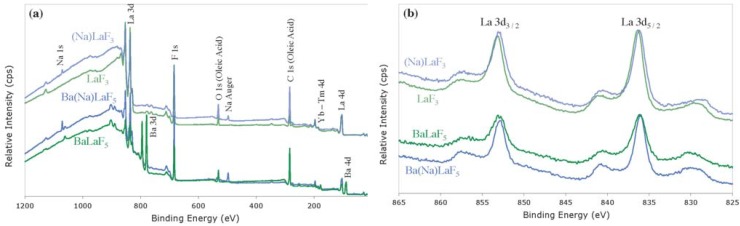
XPS survey scans of (**a1**) (Na)LaF_3_ (**pale Blue**); (**a2**) LaF_3_ (**pale Green**); (**a3**) Ba(Na)LaF_5_ (**Blue**); and (**a4**) BaLaF_5_ (**Green**) indicating the presence and absence of Na-codopant; normalized XPS La 3d spectral region (**b1**) (Na)LaF_3_ (**pale Blue**); (**b2**) LaF_3_ (**pale Green**); (**b3**) Ba(Na)LaF_5_ (**Blue**); and (**b4**) BaLaF_5_ (**Green)** indicating a shift in binding energy in La 3d_5/2_ core orbital upon Na^+^ inclusion.

XPS characterization of Na-codoped LaF_3_ and BaLaF_5_ UCNCs, compared to their undoped controls (Figure 4), reveals insights into the surface composition and local crystal field environments of the host matrices. XPS analysis and Gaussian deconvolution of the of Na 1s and La 3d orbital spectra in (Na)LaF_3_ show a low energy component indicative of interstitial ion insertion into the crystal structure, that is clearly absent in the undoped LaF_3_ control (Table 2; Figure S3) [42,44]. The presence of this low energy shoulder in both Na 1s and La 3d orbital XPS spectral regions suggests that Na^+^ inclusion contributes to La^3+^ substitution, as evidenced by the presence of interstitial La^3+^ components. The Na 1s line also displays a low binding energy, interstitial component, though less in contribution (2.65%) in total Na^+^ incorporation *versus* La^3+^ substitution (~97%). Analysis and deconvolution of the Na 1s, La 3d, and Ba 3d orbital lines of Ba(Na)LaF_5_ display a low energy spectral shoulder exhibited in the Ba 3d orbital alone, indicating direct substitution of Na^+^ for Ba^2+^ and interstitial Ba^2+^ inclusion, with no observed La^3+^ substitution (Table 2).

**Table 1:** Inductively coupled plasma optical emission spectroscopy (ICP-OES) compositional analysis of 0.5%Tm, 20%Yb codoped (Na)LaF_3_, and Ba(Na)LaF_5_ upconverting nanocrystals (UCNC) and core@shell materials as total ions detected (mol%/L) and ratios of components *vs*. total rare earth (R.E.) ions detected (%). Note: potassium (K^+^) content was measured in trace (<0.3 mol%/L) amounts or not detected (ND).

	**Conc. Na**	**Conc. Ba**	**Conc. La**	**Conc. Tm**	**Conc. Yb**	**Conc. Ratio**
	**mol%/L**	**mol%/L**	**mol%/L**	**mol%/L**	**mol%/L**	**(Yb/Tm): La**
**(Na)LaF_3_ Core**	22.15	ND	62.11	0.38	15.36	0.26
**(Na)LaF_3_ Core@Shell**	19.02	ND	67.09	0.34	13.55	0.12
**Ba(Na)LaF_5_ Core**	25.68	31.58	33.99	0.21	8.54	0.25
**Ba(Na)LaF_5_ Core@Shell**	12.82	25.02	55.59	0.18	6.38	0.21
	**Na:RE (%)**	**Ba:RE (%)**	**La:RE (%)**	**Tm:RE (%)**	**Yb:RE (%)**	**Sum mol% RE**
**(Na)LaF_3_ Core**	28.45	ND	79.78	0.49	19.73	77.85
**(Na)LaF_3_ Core@Shell**	23.48	ND	82.85	*0.42*	*16.73*	80.98
**Ba(Na)LaF_5_ Core**	60.08	73.89	79.53	0.49	19.98	42.74
**Ba(Na)LaF_5_ Core@Shell**	20.63	40.26	89.45	*0.29*	*10.27*	62.15

**Table 2:** X-ray photoelectron spectroscopy (XPS) Na 1s, La 3d, and Ba 3d regions scans binding energies (eV) in (Na)LaF_3_, LaF_3_, Ba(Na)LaF_5_, and BaLaF_5_. In cases of multicomponent Gaussian fits, percentage values listed correspond to the extent of ion incorporation by substitution (primary peak) and insertion into interstitial sites (lower energy component). ND denotes Not Detectable.

Regions (fitted):	(Na)LaF_3_	LaF_3_	Ba(Na)LaF_5_	BaLaF_5_
Na 1s	1071.25 eV (97.35%)	ND	1071.25 eV	ND
Na 1s interstitial	1069.2 eV (2.65%)	ND	ND	ND
La 3d_3/2_	852.94 eV (38.64%)	853.21 eV	852.87 eV	852.87 eV
La 3d_3/2_ interstitial	851.44 eV (1.23%)	ND	ND	ND
La 3d_5/2_	836.134 eV (58.71%)	836.38 eV	836.04 eV	836.12 eV
La 3d_5/2_ interstitial	834.16 eV (1.43%)	ND	ND	ND
Ba 3d_3/2_	ND	ND	794.94 eV (38.94%)	795.07 eV
Ba 3d_3/2_ interstitial	ND	ND	793.77 eV (1.55%)	ND
Ba 3d_5/2_	ND	ND	779.64 eV (58.75%)	779.77 eV
Ba 3d_5/2_ interstitial	ND	ND	778.21 eV (0.77%)	ND

The effect of substitution of both Na^+^ into LaF_3_ and BaLaF_5_ and charged ions in interstitial sites proximal to R.E.^3+^ ions, is seen in the XPS spectra of (Na)LaF_3_ and Ba(Na)LaF_5_ as a decrease in binding energy of the La 3d_5/2_ orbital line. This global decrease in La 3d_5/2_ binding energy upon Na-codoping denotes perturbation in the local crystal field environment surrounding rare earth emitting ions and an overall decrease in local field symmetry [19,40,42,44]. This reduction in symmetry leads to increasingly allowed R.E.^3+^-R.E.^3+^ energy transfer UC processes and emissive transitions that are formally spin and Laporte forbidden, which in turn result in Tm^3+^ luminescence enhancement [12].

### 2.3. Core@Shell Compositional Analysis

Shells of (Na)LaF_3_ and Ba(Na)LaF_5_, without Tm/Yb dopants, were solvothermally grown, sequentially following UCNC core synthesis in the same Teflon^®^-lined autoclavable bombs (see Section 3.1) in the interest of further enhancing dominant UV emissions [38]. The conditions utilized resulted in radial growth of 7.5 nm (Na)LaF_3_ and 1.1 nm Ba(Na)LaF_5_ shells, respectively, on their same host material cores as measured from TEM images (Figure 1). Conditions for Ba(Na)LaF_5_ shelling were conducted at a low Ba(NO_3_)_2_ concentration in order to avoid formation of BaF_2_ shells; thicknesses of Ba(Na)LaF_5_ shells were thus significantly reduced relative to those of corresponding (Na)LaF_3_ shells, and resulted in diminished emission enhancements that derive from reduction in surface defects near emitting ions or augmentation of distance between emitting ions and solvent. Decreased Yb(Tm):La ratios detected in ICP-OES of dissolved core@shell samples (Table 3), in tandem with consistent particle monodispersity (see TEM data, Figure 1), are consistent with UCNC shelling. Analysis of the Yb(Tm):La ratios of the surfaces of the core@shell nanomaterials is far more indicative of effective shelling. XPS probes the surface compositions of these materials (between 4 and 6 nm) and analysis of Yb(Tm):La ratios on the surface of core@shell materials show a marked reduction in the Yb(Tm):La molar ratio compared to that of their UCNC core counterparts. Congruent with expectations of XPS *versus* ICP-OES compositional analysis, XPS data evince a reduction in the Yb(Tm):La molar ratio relative to that determined via ICP-OES, as XPS interrogates ion composition within 4–6 nm of the surface. These experiments signal discrete shell formation under these solvothermal synthetic conditions, as redissolution of UCNC cores and redistribution of emitting ions in the shell layer would give rise to similar Yb(Tm):La ratios in the ICP-OES and XPS experiments. In the Ba(Na)LaF_5_ core@shell *versus* UCNC core material, ICP-OES shows a minimal change in Yb(Tm):La ratio, due to the addition of a thin shell (~1 nm), whereas XPS composition analysis shows a significant reduction in Yb(Tm):La ratio (0.5:1), indicating the presence of a distinct shell composition.

It should also be noted that solvothermal shelling permits secondary, thermodynamically-driven, recrystallization processes to occur in restructuring of the UCNCs to a more stable crystallographic phase [1,32,45,46]. In the (Na)LaF_3_ core@shell nanomaterial, XRD indicates that β-NaYbF_4_ is present (Figure 3) as a minor inclusion and β-NaLaF_4_ appears in the Ba(Na)LaF_5_ core@shell product. Though binding energy shifts were undetected in the XPS spectrum of the Yb 4d region, a noteworthy increase in the NIR ^3^H_4_ → ^3^H_6_ (803 nm) Tm^3+^ transition intensity in (Na)LaF_3_ core@shell material is observed relative to (Na)LaF_3_ UCNC cores (Figure 2a), consistent with highly efficient Yb-to-Tm ETU processes and dominant NIR emission previously reported for Tm^3+^ doped, NaYbF_4_ [7]. This topic is further discussed with respect to UC mechanism considerations in Section 2.4, Power Dependence.

**Table 3:** XPS survey scans of (Na)LaF_3_, (Na)LaF_3_ core@shell, Ba(Na)LaF_5_, and Ba(Na)LaF_5_ core@shell surface compositions (% element detected) and Yb/Tm:La ratio. Yb and Tm 4d lines were integrated together.

XPS Survey Scan	Na (%)	Ba (%)	La (%)	Yb/Tm (%)	F (%)	Yb/Tm: La
(Na)LaF_3_ Core	3.43	ND	13.9	1.37	81.31	0.10
(Na)LaF_3_ Core@Shell	2.14	ND	17.32	0.61	79.92	0.04
Ba(Na)LaF_5_ Core	4.94	11.56	6.61	0.65	76.24	0.10
Ba(Na)LaF_5_ Core@Shell	0.0	8.56	13.58	0.69	77.17	0.05

### 2.4. Emission Intensity *versus* Power (IvP) Dependence Studies

Laser power dependence emission studies were conducted on (Na)LaF_3_ and Ba(Na)LaF_5_ UCNCs and their core@shell counterparts to aid in determining an upconversion mechanism consistent with the observed dominant UV emission at low laser powers (<30 W/cm^2^). Emission intensity *versus* laser power density (IvP) studies were conducted using a 980 nm CW laser with power densities varying over 2.5–100 W/cm^2^ ensuring a clear transition from low to high power regimes, indicated by saturation of one or more emissive states [12,13,15]. As described by Pollnau *et al*. [47], and elaborated on by Suyver *et al*. [48], the energy transfer upconversion (ETU) mechanism, characterized by sequential energy transfers from a Yb^3+^ sensitizer to activator emitting ion (e.g., Tm^3+^), can be elucidated on a per excited state basis in the ln-ln plot of emission intensity (arbitrary units) *versus* laser power density (W/cm^2^). In the low power regime of the ln-ln IvP plot, the slopes of the linear fits of each emissive transition provide insight into a number of photons or ETU events required to populate the emitting states via a standard Yb-to-Tm sequential ETU process, in the limit where no emitting state achieves saturation, wherein the upconversion process dominates over relaxation via radiative or non-radiative decay [12,47].

**Figure 5:**
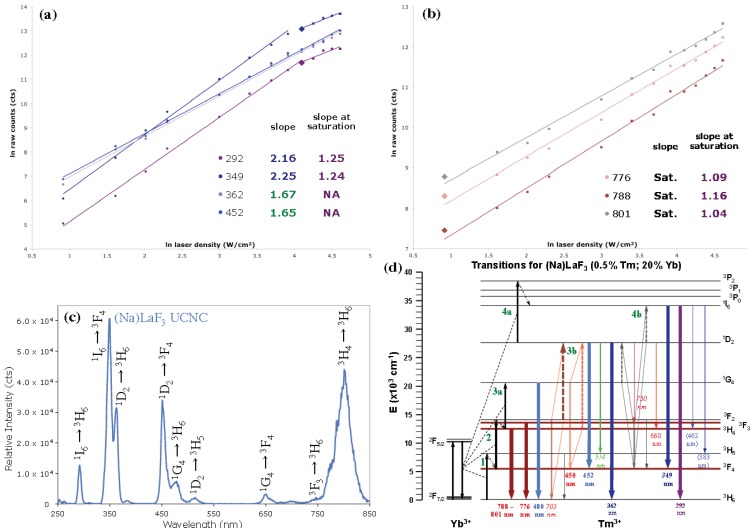
Emission intensity *vs*. 980 nm laser power density (W/cm^2^), ln-ln IvP plot of 0.5%Tm, 20%Yb codoped (Na)LaF_3_ UCNC in (**a**) UV/blue and (**b**) NIR regime, noting low power saturation of Tm^3+^ ^3^F_3_ and ^3^H_4_ states; (**c**) Emission spectrum at 20 W/cm^2^ and (**d**) ETU transition diagram for Yb^3+^-Tm^3+^. Experimental conditions: 1 mg/mL solutions in toluene at 23 °C; 980 nm CW laser excitation varying from 2.5 to 100 W/cm^2^.

Literature precedent demonstrates 2–5 photon ETU processes across Tm^3+^ 4f-4f transitions, often involving a Tm-Tm centered cross-relaxation (Tm-Tm CR) event to by-pass the ^1^G_4_ to ^1^D_2_ energy level gap [17,30], an energy gap too small to be successfully bridged with a 10,200 cm^−1^ ETU from Yb coupled to a several thousand wavenumber phonon mode, which would otherwise provide for non-radiative decay of lower energy f-block states (Figure 5d) [49]. Even under the low laser power densities utilized, the expected two photon ETU processes required to populate the ^3^H_4_ and ^3^F_3_ states are linearly fit with a slope of ~1, indicating saturation of these states even at power densities as low as 2.5 W/cm^2^. It has been suggested that core lattice defects of small UCNCs may promote CR events to occur under condition of low laser density (<30 W/cm^2^) [15], a defect condition intrinsic to Na-codoped UCNCs of sub-10 nm size. The consequences of this initial saturation of NIR emitting states in the ETU mechanism are immediately apparent in global slope reductions in each higher energy transition, shown in the ln-ln IvP plot (Figure 5). The significantly reduced slope, showing only dependence on Yb sensitizer excited state population density [47,48], serves to place Tm^3+^, when ^3^H_4_ and ^3^F_3_ states are under saturation, in a co-sensitizer role for activation of higher energy Tm^3+^ excited states (Figure 5b) [48]. From this initial condition, a single-photon, Yb-to-Tm ETU event may populate the ^1^G_4_ state, permitting ^1^G_4_ → ^3^F_4_:^3^H_4_ → ^1^D_2_ Tm-Tm CR followed by another sequential Yb-to-Tm ETU to yield a population in the 292- and 349-nm emitting, ^1^I_6_ state. Similar Tm-Tm CR of ^3^F_2_ → ^3^H_6_:^3^F_2_ → ^1^D_2_ serves to provide a secondary route to high energy UV emitting states with diminished ln-ln IvP slopes of ~2, suggesting 2 photon Yb-to-Tm ETU processes (Figure 5a). From a combination of these Tm-Tm CR events, UV emission increases in intensity as saturation of NIR emitting ^3^F_3_ and ^3^H_4_ states is achieved, invariably, to a ln-ln IvP slope of 1, congruent with the diminished radiative output of these states due to their roles in CR. Under higher laser densities (30–100 W/cm^2^), transitions originating from the ^1^I_6_ state exhibit significantly reduced slopes compared to ^1^G_4_ and ^1^D_2_ derived visible transitions, consistent with relaxation of these states through Tm-Tm CR (^1^G_4_ → ^3^F_4_:^3^H_4_ → ^1^D_2_).

**Figure 6:**
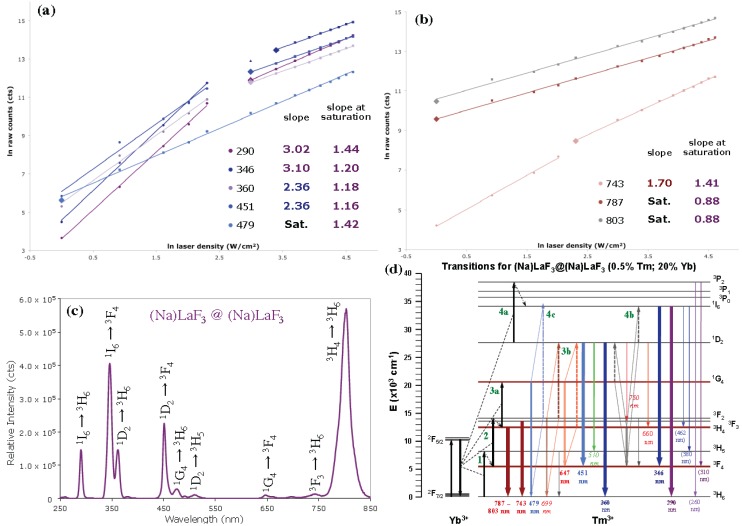
Emission intensity *vs*. 980 nm laser power density (W/cm^2^), expressed in ln-ln IvP plots of 0.5% Tm, 20% Yb codoped (Na)LaF_3_ core@shell in the (**a**) UV/blue and (**b**) NIR regimes, noting low power saturation of Tm^3+^ ^3^H_4_ state and onset of saturation (diamond markers); (**c**) Emission spectrum recorded at 20 W/cm^2^ and (**d**) ETU transition diagram of Yb^3+^-Tm^3+^. Experimental conditions: 1 mg/mL solutions in toluene at 23 °C; 980 nm CW laser excitation varying from 2.5 to 100 W/cm^2^.

The ln-ln IvP plot of the (Na)LaF_3_ core@shell system (Figure 6a,b) shows a further departure from the standard, sequential ETU model, including a low power density saturated ^1^G_4_ state (479 nm transition) in addition to a saturated ^3^H_4_ state. The onset of saturation of these UV and blue emitting states at decreased laser power densities (20–30 W/cm^2^) suggests a higher probability of a ^1^G_4_ → ^3^H_6_:^3^F_3_ → ^1^I_6_ Tm-Tm CR. Shelling of UCNCs has also been seen to increase the lifetimes of emitting states, allowing for further complex radiative and CR pathways to occur [18,36,39]. The appearance of NaYbF_4_ inclusions in the (Na)LaF_3_ core@shell as indicated in the XRD data (Figure 3a), an UCNC composition established to have a high efficiency UC mechanism into NIR emitting ^3^F_3_ and ^3^H_4_ states [5,7], serves in part to explain the significant 803 nm emission intensity seen in the ln-ln IvP plot.

Ba(Na)LaF_5_ UCNC and Ba(Na)LaF_5_ core@shell materials exhibit similar ln-ln IvP plotted slope reductions per radiative transition in the NIR emitting states in the low laser density regime (<30 W/cm^2^) as was observed for (Na)LaF_3_ UCNCs (Table 4) (see Supplemental Information for detailed ln-ln IvP plots and proposed ETU transition state diagrams). In kind, ETU processes dominate over linear radiative and non-radiative decay of intermediate ^3^F_3_ and ^3^H_4_ states, with Tm-Tm CR of NIR transitions significantly contributing in the population of higher energy excited states and providing a means of increasing UV emissive output [47]. The lack of a dominant UV emission band in Ba(Na)LaF_5_, *in lieu* of a broad band emission profile throughout the UV-vis-NIR regime (Figure 2b), may be due in part to a lack of saturation of the NIR emitting states at low laser power, thus, diminishing the probability of Tm-Tm CR as a viable mechanism for population of higher energy, UV emissive, Tm^3+^ excited states.

**Table 4:** Emission intensity *vs*. 980 nm laser power density (W/cm^2^), ln-ln IvP plotted linear slopes of 0.5%Tm, 20%Yb codoped (**a**) (Na)LaF_3_ UCNC; (**b**) (Na)LaF_3_ core@shell; (**c**) Ba(Na)LaF_5_ UCNC; and (**d**) Ba(Na)LaF_5_ core@shell; emission bands and transitions, ln-ln IvP slopes per radiative transition at low laser density, and slopes at saturation are given. Slopes in **bold**, *italics*, and plain text correspond to matched Tm states of origin. NA denotes state saturation Not Achieved.

**(a)**					**(b)**			
**(Na)LaF_3_ UCNC**	**Transition**	**slope**	**slope sat.**		**(Na)LaF_3_ core@shell**	**Transition**	**slope**	**slope sat.**
292	^1^I_6_ → ^3^H_6_	*2.16*	*1.25*		290	^1^I_6_ → ^3^H_6_	*3.02*	*1.44*
349	^1^I_6_ → ^3^F_4_	*2.25*	*1.24*		346	^1^I_6_ → ^3^F_4_	*3.10*	*1.2*
362	^1^D_2_ → ^3^H_6_	*1.67*	*NA*		360	^1^D_2_ → ^3^H_6_	*2.36*	*1.18*
452	^1^D_2_ → ^3^F_4_	*1.65*	*NA*		451	^1^D_2_ → ^3^F_4_	*2.36*	*1.16*
480	^1^G_4_ → ^3^H_6_	1.57	NA		479	^1^G_4_ → ^3^H_6_	Sat.	1.42
514	^1^D_2_ → ^3^H_5_	*1.83*	*NA*		510	^1^D_2_ → ^3^H_5_	*2.12*	*1.41*
650	^1^G_4_ → ^3^F_4_	1.61	NA		647	^1^G_4_ → ^3^F_4_	1.63	1.37
776	^3^F_3_ → ^3^H_6_	Sat.	1.09		743	^3^F_3_ → ^3^H_6_	1.70	1.41
788	^3^H_4_ → ^3^H_6_	Sat.	1.16		787	^3^H_4_ → ^3^H_6_	Sat.	0.88
801	^3^H_4_ → ^3^H_6_	Sat.	1.04		803	^3^H_4_ → ^3^H_6_	Sat.	0.88
**(c)**					**(d)**			
**Ba(Na)LaF_5_ UCNC**	**Transition**	**slope**	**slope sat.**		**Ba(Na)LaF_5_ core@shell**	**Transition**	**slope**	**slope sat.**
292	^1^I_6_ → ^3^H_6_	*2.37*	*1.38*		292	^1^I_6_ → ^3^H_6_	*2.21*	*NA*
349	^1^I_6_ → ^3^F_4_	*2.38*	*1.36*		349	^1^I_6_ → ^3^F_4_	*2.28*	*1.38*
362	^1^D_2_ → ^3^H_6_	*1.69*	*NA*		362	^1^D_2_ → ^3^H_6_	*1.71*	*NA*
452	^1^D_2_ → ^3^F_4_	*1.69*	*NA*		452	^1^D_2_ → ^3^F_4_	*1.65*	*NA*
463	^1^D_2_ → ^3^F_4_	*1.63*	*NA*		463	^1^D_2_ → ^3^F_4_	*1.77*	*NA*
480	^1^G_4_ → ^3^H_6_	1.49	NA		480	^1^G_4_ → ^3^H_6_	1.60	NA
515	^1^D_2_ → ^3^H_5_	*1.70*	*NA*		515	^1^D_2_ → ^3^H_5_	*1.91*	*NA*
650	^1^G_4_ → ^3^F_4_	1.32	NA		650	^1^G_4_ → ^3^F_4_	1.63	NA
772	^3^F_3_ → ^3^H_6_	1.54	NA		772	^3^F_3_ → ^3^H_6_	1.50	NA
788	^3^H_4_ → ^3^H_6_	1.24	NA		788	^3^H_4_ → ^3^H_6_	1.27	NA

## 3. Experimental Section

### 3.1. Synthesis

#### 3.1.1. Materials

La(NO_3_)_3_·6H_2_O (99.999%), Yb(NO_3_)_3_·5H_2_O (99.999%), Tm(NO_3_)_3_·5H_2_O (99.9%), Ba(NO_3_)_2_ (99.95%), KF (99.99%), NaOH (99.99%), and NH_4_F (>99.99%) were purchased from Sigma Aldrich (St. Louis, MO, USA) and used without further purification. Oleic acid (>97%), water (Optima grade), and NH_4_OH (Certified ACS Plus) were purchased from Fisher Scientific (Pittsburgh, PA, USA). Ethanol (200 proof, anhydrous, USP) was purchased from KOPTEC (King of Prussia, PA, USA).

Teflon^®^ autoclave sleeves (120 mL) were soaked overnight in a base bath of 2-propanol and potassium hydroxide (saturated), rinsed with distilled water, subjected to aqua regia (3:1 HCl:HNO_3_; ACS Grade, Fisher Scientific (Pittsburgh, PA, USA)) for 30 min, rinsed again with distilled water, then treated again under base bath for an additional 20 min. The Teflon^®^ sleeves were subsequently rinsed thoroughly with distilled water and ethanol and left to dry in air prior to use.

#### 3.1.2. Na-Doped LaF_3_ Synthesis

Following the solvothermal procedure of Wang *et al*. [34], 2.4 g (0.06 mol) NaOH, 4 mL Optima grade water, 20 mL ethanol, and 38.1 mL (0.12 mol) oleic acid were added in sequential order to a round-bottomed flask under vigorous stirring. The mixture was occasionally warmed with a heat gun to facilitate dissolution. Lanthanide nitrate hydrates (1 mmol) at a molar ratio of 0.795 La:0.20 Yb:0.005 Tm in 2 mL Optima grade water were added in one portion and stirred until fully dissolved, followed by KF (174.3 mg, 3 mmol) in 2 mL Optima grade water. Solutions were stirred for 30 min before being transferred into 120 mL Teflon^®^ sleeves, sealed in stainless steel autoclavable bombs, and placed in an oven at 200 °C for 20 h. Once cooled to room temperature, the particles were precipitated with a mixture of 60 mL ethanol and 40 mL *n*-hexane and subjected to centrifugation at 14,000 rpm for 20 min at 5 °C. The particles were then resuspended in 30 mL n-hexane and 2 mL water with sonication and precipitated with 28 mL ethanol, centrifuged at 14,000 rpm for 20 min at 5 °C. This step was followed by a 50% ethanol/water wash and a final ethanol wash, both run at 14,000 rpm for 10 min at 5 °C.

#### 3.1.3. Na-Doped BaLaF_5_ Synthesis

Following the solvothermal procedure of Wang *et al*. [34], 2.4 g (0.06 mol) NaOH, 4 mL Optima grade water, 20 mL ethanol, and 38.1 mL (0.12 mol) oleic acid were added in sequential order to a round-bottomed flask under vigorous stirring. The mixture was occasionally warmed with a heat gun to facilitate dissolution. Barium nitrate (1 mmol) and lanthanide nitrate hydrates (1 mmol) at a molar ratio of 0.795 La:0.20 Yb:0.005 Tm in 2 mL Optima grade water were added in one portion and stirred until fully dissolved, followed by KF (290.5 mg, 5 mmol) in 2 mL Optima grade water. Solutions were stirred for 30 min before being transferred into 120 mL Teflon^®^ sleeves, sealed in stainless steel autoclavable bombs, and placed in an oven at 200 °C for 20 h. Once cooled to room temperature, the particles were precipitated with a mixture of 60 mL ethanol and 40 mL *n*-hexane and subjected to centrifugation at 14,000 rpm for 20 min at 5 °C. The particles were then resuspended in 30 mL *n*-hexane and 2 mL water with sonication and precipitated with 28 mL ethanol, centrifuged at 14,000 rpm for 20 min at 5 °C. This step was followed by a 50% ethanol/water wash and a final ethanol wash, both run at 14,000 rpm for 10 min at 5 °C.

#### 3.1.4. (Na)LaF_3_@(Na)LaF_3_ and Ba(Na)LaF_5_@Ba(Na)LaF_5_ Core/Shell Syntheses

Na-doped LaF_3_ or BaLaF_5_ cores were made according to the solvothermal method outlined above, and shells of the same materials were grown following a modified procedure from Zhang *et al*. [38]. Excess KF, 232.4 mg (4 mmol) for a (Na)LaF_3_ shell synthesis or 387.5 mg (6.67 mmol) for a Ba(Na)LaF_5_ shell synthesis, was added to the solvothermal preparations of core nanocrystals. After 20 h in the oven at 200 °C for core synthesis, the solution was allowed to cool to room temperature, then stirred vigorously for 30 min. La(NO_3_)_3_·6H_2_O (433.01 mg, 1 mmol) or La(NO_3_)_3_·6H_2_O (1 mmol) and 87.03 mg (0.33 mmol) Ba(NO_3_)_2_ were dissolved in a 3 mL solution of Optima grade water which was added dropwise, followed by stirring for an additional 30 min. The autoclaves were resealed and placed in an oven at 200 °C for an additioanl 3 h, cooled to room temperature, and the reaction mixtures purified following the above procedure for BaLaF_5_ core precipitation.

### 3.2. Characterization

Transmission electron microscopy (TEM) images were acquired using a FEI Tecnai G2 Twin microscope (FEI, Hillsboro, OR, USA) operated at 200 kV and equipped with a TIA digital camera to obtain data on particle morphology, size, and distribution. The UCNCs were dispersed in 1 mg/mL toluene solutions, drop cast, and dried on copper grids (200 mesh) with carbon/formvar support grids.

X-ray powder diffraction (XRD) patterns and phase analysis of the particles were obtained using a Philips Panalytical X’Pert PRO MRD HR X-ray diffraction system (PANalytical, Westborough, MA, USA) with Cu Kα source at 1.5405 Å and pressed powder samples.

Emission and ln-ln IvP plotted spectra were acquired using an Edinburgh FLS920 system (Edinburgh Instruments, Livingston, UK) at 4 nm slit width, with a 980 nm continuous wave diode laser focused to a 320 μm diameter spot size, measured by the razor-edge method at the 90/10 diameter in 5 µm steps. Spectra were recorded over a 250–850 nm spectral range (1 nm steps; integration time = 0.5 s) and corrected for variations in detector response as a function of wavelength using correction files supplied by the manufacturer. All samples were run at concentrations of 1 mg/mL in toluene at 23 °C.

Inductively coupled plasma optical emission spectrometry (ICP-OES) was performed with a Perkin Elmer Optima 2000 DV system (Perkin Elmer, Waltham, MA, USA) on UCNC samples following treatment with boric acid and dissolution in aqua regia; diluted with water 10–20×.

X-ray photoelectron spectroscopy (XPS) was also performed using a Kratos Analytical Axis Ultra system (Kratos Analytical, Wharfside, UK) using a Mono (Al) emission source and charge neutralizer. Pressed powder samples were affixed to copper tape. Regions scans were collected at 0.1 eV steps with a minimum of 3 sweeps to determine binding energies of individual elements. Survey scans were collected at 1 eV steps with 3 sweeps to determine elemental composition. The resulting spectra were deconvolved, fit, and integrated using CasaXPS.

## 4. Conclusions

We report the synthesis and characterization of sub-10 nm, dominant UV emissive, Na-codoped LaF_3_ and BaLaF_5_ UCNCs and their core@shell derivatives, generated via a one-pot solvothermal approach. For all materials, Na-codopant of approximately 20% (*versus* total dissolved cation concentration) was present and determined to play a significant role in the one- to two-order of magnitude enhancement of Yb-to-Tm ETU *vs*. non-doped 0.5% Tm, 20% Yb samples in the same size regime. XRD analysis of (Na)LaF_3_ exhibited 2θ value diminution of all crystallographic peaks, indicating an increase in unit cell volume; XPS spectra of La 3d and Na 1s regions revealed a marked decrease in La 3d binding energy (eV) and low energy peak components, showing relocation of La^3+^ into interstitial sites for charge balancing upon La-to-Na substitution. These modifications in crystallographic dimensions and emitting ion environment are consistent with a reduction in local crystal field symmetry and the observed enhancement of UC luminescence. Na-codoping into the BaLaF_5_ host matrix showed an increase in all 2θ values and an associated reduction in unit cell volume (XRD). XPS La and Ba 3d regions scans showed only single Gaussian fits in both BaLaF_5_ and Ba(Na)LaF_5_, indicating a reduction in La 3d binding energy and no substitution with La^3+^. Though the relative contributions of change in lattice volumes with respect to shifts in rare earth element binding energies cannot be quantified in relation to emission enhancement, they are known to decrease the local crystal field symmetry around sensitizer-activator pairs and serve to enhance UC processes.

(Na)LaF_3_ and Ba(Na)LaF_5_ UCNCs and their core@shell derivatives were subjected to emission intensity *vs*. laser power dependence studies (IvP) to mark deviations from known upconversion mechanisms. In all cases, significant slope reductions in the ln-ln IvP plots were noted, and in the case of (Na)LaF_3_ and its core@shell derivative, NIR emitting ^3^F_3_ and ^3^H_4_ states were found populated to saturation even at the lowest laser power densities tested (2.5 W/cm^2^). The ln-ln IvP plot linear slope reductions, as an initial condition of ^3^H_4_ saturation, are surmised to report an increase Tm-Tm cross-relaxation processes that promote excited state populations in UV emitting ^1^I_6_ and ^1^D_2_ states, exhibiting depressed slopes and virtual 2–3 photon ETU events. Further analysis of these exemplary, dominant UV emissive nanomaterials is warranted in the pursuit of UCNCs with specific emission band enhancement and unique emission signatures.

## Supplementary Materials

Supplementary File 1
