# Supplementary material for: One-Pot Solvothermal Synthesis of Highly Emissive, Sodium-Codoped, LaF3 and BaLaF5 Core-Shell Upconverting Nanocrystals
Source: Nanomaterials (Basel). 2014 Jan 8;4(1):69–86. doi: 10.3390/nano4010069 (PMC5304608; doi:10.3390/nano4010069)
Supplement: Supplementary File 1 [file nanomaterials-04-00069-s002.pdf]

Supplemental Information

S1. Experimental Section

S1.1 Synthesis

S1.1.1 Materials

$La(NO_3)_3·6H_2O$ (99.999%), $Yb(NO_3)_3·5H_2O$ (99.999%), and $Tm(NO_3)_3·5H_2O$ (99.9%), $Ba(NO_3)_2$ (99.95%), NH_4F (>99.99%), and sodium trifluoroacetate (≥99.0%) were purchased from Sigma Aldrich (St. Louis, MO, USA) and used without further purification. Oleic acid (>97%), water (Optima grade), NH_4OH (Certified ACS Plus), and trifluoroacetic acid (≥99.5%) were purchased from Fisher Scientific (Pittsburgh, PA, USA). Ethanol (200 proof, anhydrous, USP) was purchased from KOPTEC (King of Prussia, PA, USA). Rare earth trifluoroacetates were prepared as per Hara *et al.* [1] using rare earth oxides purchased from Alfa Aesar (Ward Hill, MA, USA) of no less than 99.997% purity. Barium trifluoroacetate was prepared in the same fashion using $BaCO_3$ (99.997%; Alfa Aesar).

Teflon autoclave sleeves (120 mL) were soaked overnight in a base bath of 2-propanol and potassium hydroxide, rinsed with distilled water, subjected to aqua regia (3:1 $HCl:HNO_3$) for 30 min, rinsed again with distilled water, then treated again under base bath for an additional 20 min. The sleeves were subsequently rinsed thoroughly with distilled water and ethanol and left to dry in air prior to use.

S1.1.2 LaF_3 Synthesis

LaF_3 containing no sodium dopant was generated in similar fashion to the Na-doped LaF_3 synthesis [2] with substitution of NaOH for NH_4OH (2.36 mL, 0.035 mol, 14.8 N soln.) and KF for NH_4F (111.11 mg, 3 mmol). All other reagent concentrations and reaction and purification conditions remained the same.

S1.1.3 $BaLaF_5$ Synthesis

Following a modified thermal decomposition approach of Vetrone *et al.* [3], to a clean, oven dried, 100 mL, 2-neck roundbottom flask with magnetic stir bar, thermocouple, and affixed reflux condenser was added 5.08 mg $Tm(TFA)_3$ (0.010 mmol), 204.83 mg $Yb(TFA)_3$ (0.40 mmol), 759.92 mg $La(TFA)_3$ (1.59 mmol), 726.70 mg $Ba(TFA)_2$ (2.00 mmol), 20 mL 1-Octadecene (90%, Sigma-Aldrich, St. Louis, MO, USA), and 20 mL Oleic acid. The apparatus was evacuated (<15 mtorr) and stirred at 125 °C for 45 min to remove residual water then sparged with argon for 20 min. The reaction apparatus was then transferred to a molten eutectic salt bath and the reaction heated over a 7 min ramp to an internal temperature of 315 °C and held at temperature for 1 h under argon. The reaction was then cooled to RT, poured into an equivalent volume of absolute ethanol, sonicated, and centrifuged at 21k rcf (~14k RPM) for 20 min. The resulting pellet was resuspended and centrifuged in similar fashion with 50:50 *n*-hexane:ethanol, followed by a wash of 50:50; water:ethanol, and a final wash of absolute ethanol. The purified nanocrystals were then dried in air overnight.

S2. Results and Discussion

Figure S1. Transmission Electron Microscopy (TEM) images of 0.5%Tm, 20%Yb codoped UCNCs in host lattices (**a**) (Na)LaF_3 [80 kx, 100 nm scale bar]; (**b**) (Na)LaF_3 [700 kx, 10 nm scale bar]; (**c**) Ba(Na)LaF_5 [80 kx, 100 nm scale bar]; (**d**) Ba(Na)LaF_5 [700 kx, 10 nm scale bar]; (**e**) (Na)LaF_3 core@shell [80 kx, 100 nm scale bar]; (**f**) (Na)LaF_3 core@shell [700 kx, 10 nm scale bar]; (**g**) Ba(Na)LaF_5 core@shell [200 kx, 50 nm scale bar]; (**h**) Ba(Na)LaF_5 core@shell [700 kx, 10 nm scale bar].

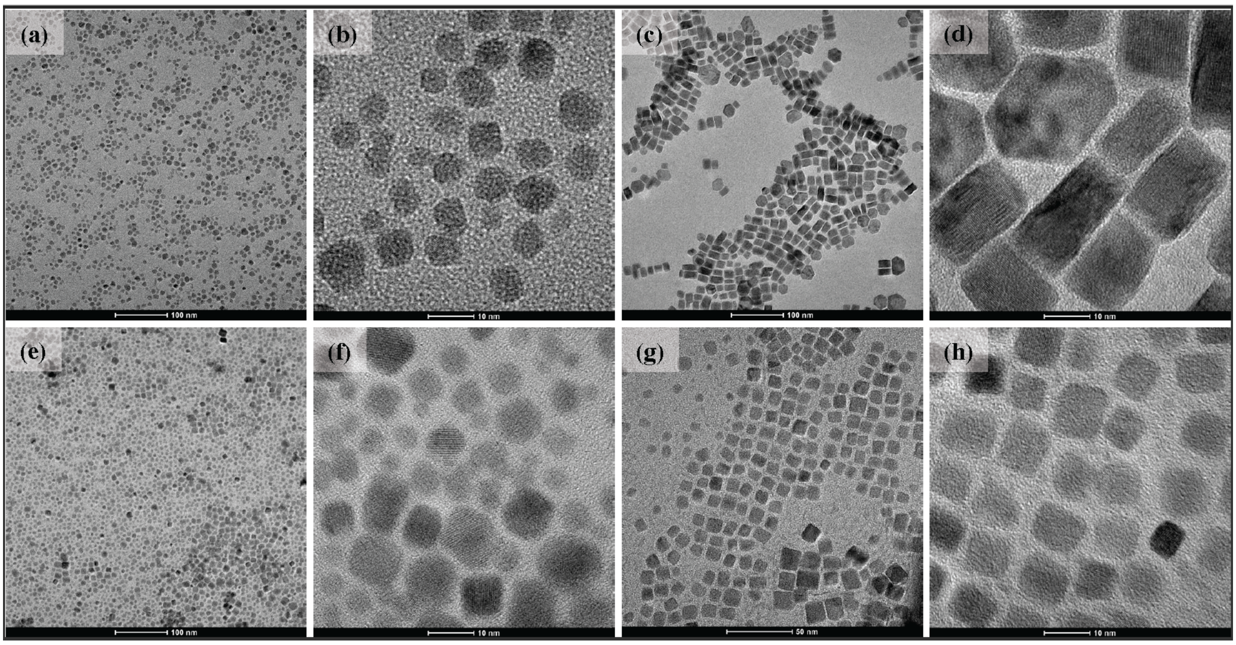

Figure S2. Transmission Electron Microscopy (TEM) images of 0.5%Tm, 20%Yb codoped UCNCs in host lattices (**a**) LaF_3 [80 kx, 100 nm scale bar], (**b**) LaF_3 [700 kx, 10 nm scale bar], (**c**) $BaLaF_5$ [80 kx, 100 nm scale bar], (**d**) $BaLaF_5$ [700 kx, 10 nm scale bar].

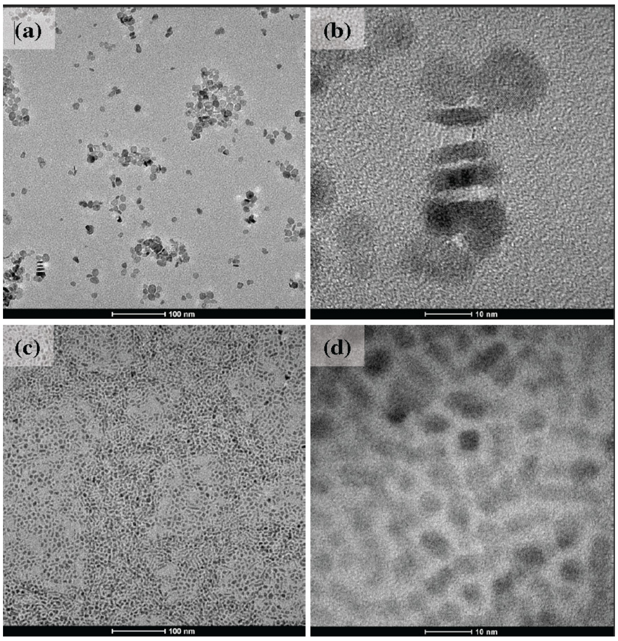

Table S1. X-Ray Powder Diffractions (XRD) 2θ (°) values of (**a1**) LaF_3 [**pale Green**]; (**a2**) $(Na)LaF_3$ core [**pale Blue**]; (**a3**) $(Na)LaF_3@(Na)LaF_3$ [**pale Violet**]; (**b1**) $BaLaF_5$ [**Green**]; (**b2**) $Ba(Na)LaF_5$ core [**Blue**]; (**b3**) $Ba(Na)LaF_5@Ba(Na)LaF_3$ [**Violet**]; associated Miller indices (hkl).

[h k l]	LaF_3	$(Na)LaF_3$ Core	$(Na)LaF_3$ Core@Shell
[002] & [110]	24.95°	24.83°	24.29°
[111]	27.85°	27.49°	27.73°
[300]	44.15°	43.85°	43.83°
[113]	45.07°	44.55°	45.05°
[302]	51.09°	51.11°	50.81°
[221]	53.03°	52.83°	52.77°
**[h k l]**	**$BaLaF_5$**	**$Ba(Na)LaF_5$ Core**	**$Ba(Na)LaF_5$ Core@Shell**
[111]	25.05°	25.87°	25.97°
[200]	29.33°	29.91°	30.05°
[220]	42.69°	42.91°	43.01°
[311]	50.25°	50.71°	50.91°
[222]	NA	53.07°	53.37°

Figure S3. XPS region scans of $(Na)LaF_3$ [Na 1s], $(Na)LaF_3$ [La 3d], LaF_3 [La 3d], $Ba(Na)LaF_5$ [Na 1s], $Ba(Na)LaF_5$ [La 3d], and $Ba(Na)LaF_5$ [Ba 3d].

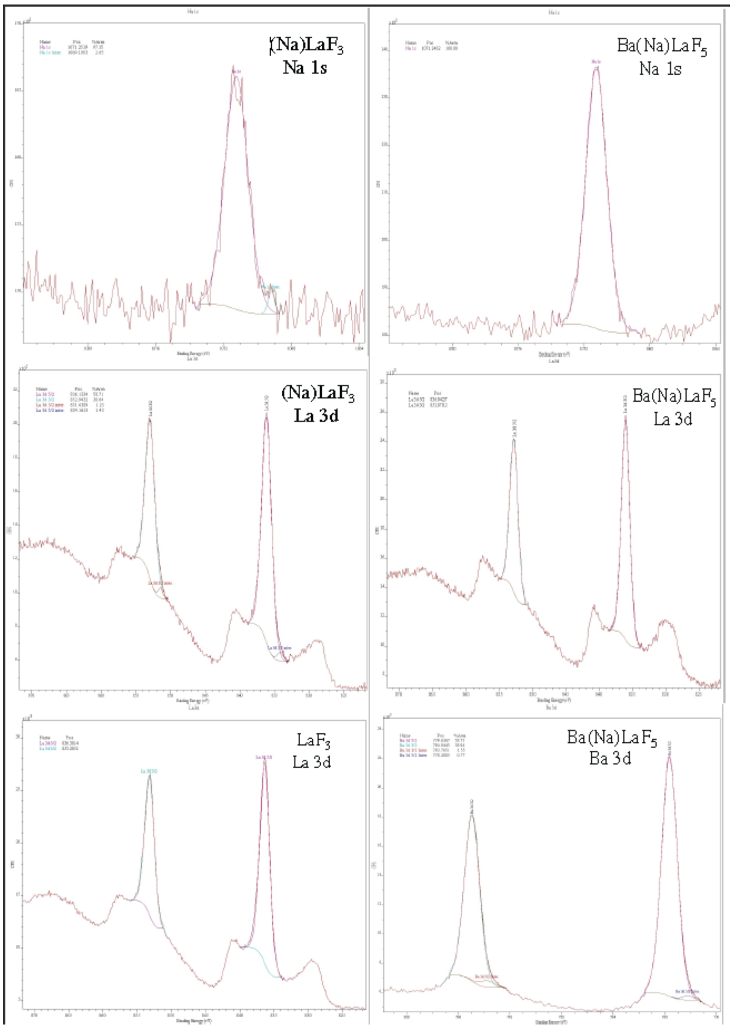

Figure S4. Emission intensity *vs.* 980 nm laser power density (W/cm^2), ln-ln IvP plot of 0.5%Tm, 20%Yb codoped (Na)LaF$_3$ UCNC in (**a**) UV/blue; (**b**) NIR; and (**c**) Vis regime and (**d**) ETU transition diagram of Yb^{3+}-Tm^{3+}. Experimental conditions: 1 mg/mL solutions in toluene at 23 °C; 980 nm CW laser excitation varying from 2.5 to 100 W/cm^2.

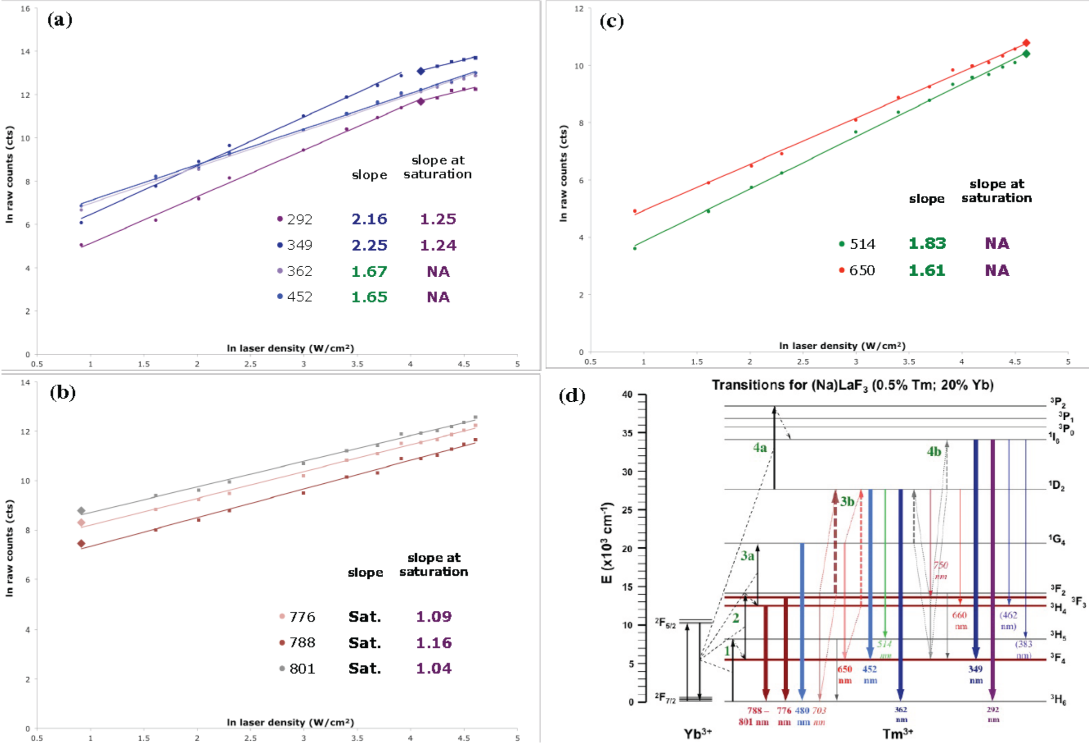

Figure S5. Emission intensity *vs.* 980 nm laser power density (W/cm^2), ln-ln IvP plot of 0.5%Tm, 20%Yb codoped (Na)LaF$_3$ core@shell in (**a**) UV/blue; (**b**) NIR; and (**c**) Vis regime and (**d**) ETU transition diagram of Yb^{3+}-Tm^{3+}. Experimental conditions: 1 mg/mL solutions in toluene at 23 °C; 980 nm CW laser excitation varying from 2.5 to 100 W/cm^2.

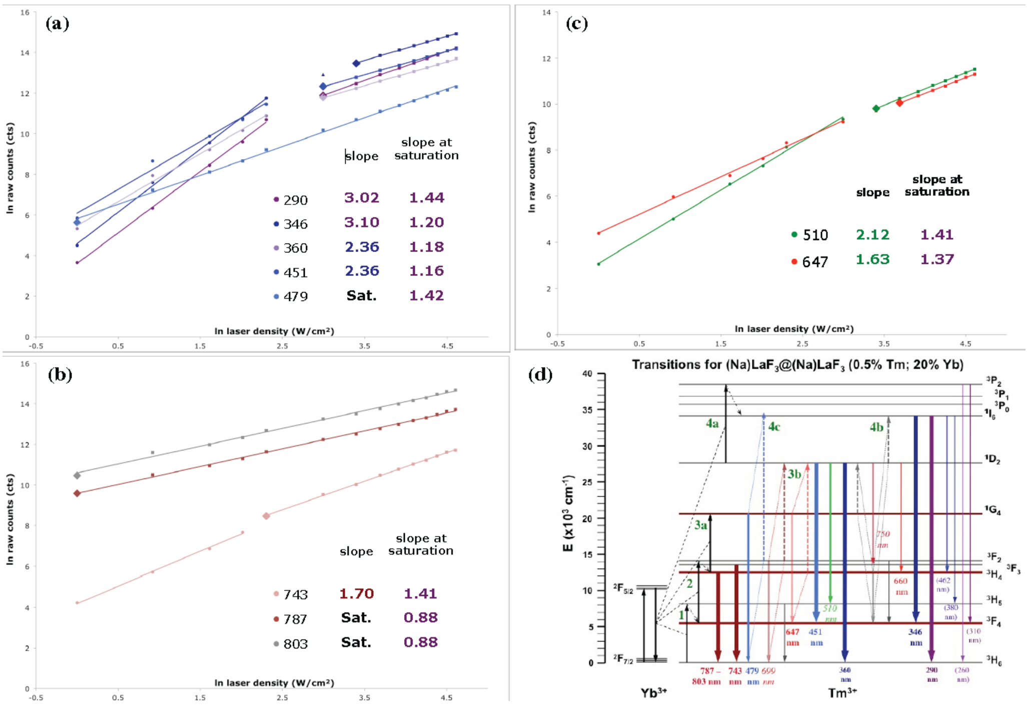

Figure S6. Emission intensity *vs*. 980 nm laser power density (W/cm^2), ln-ln IvP plot of 0.5%Tm, 20%Yb codoped Ba(Na)LaF$_5$ UCNC in (**a**) UV/blue; (**b**) NIR; and (**c**) Vis regime and (**d**) ETU transition diagram of Yb^{3+}-Tm^{3+}. Experimental conditions: 1 mg/mL solutions in toluene at 23 °C; 980 nm CW laser excitation varying from 2.5 to 100 W/cm^2.

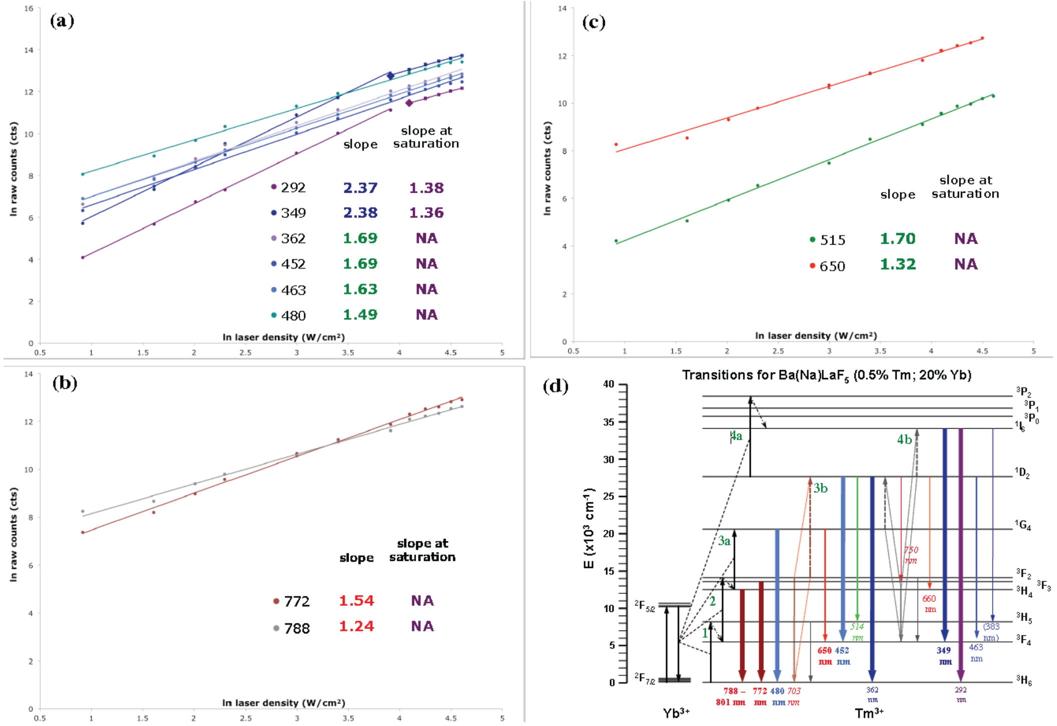

Figure S7. Emission intensity *vs*. 980 nm laser power density (W/cm^2), ln-ln IvP plot of 0.5% Tm, 20% Yb codoped Ba(Na)LaF$_5$ core@shell in (**a**) UV/blue; (**b**) NIR; and (**c**) Vis regime and (**d**) ETU transition diagram of Yb^{3+}-Tm^{3+}. Experimental conditions: 1 mg/mL solutions in toluene at 23 °C; 980 nm CW laser excitation varying from 2.5 to 100 W/cm^2.

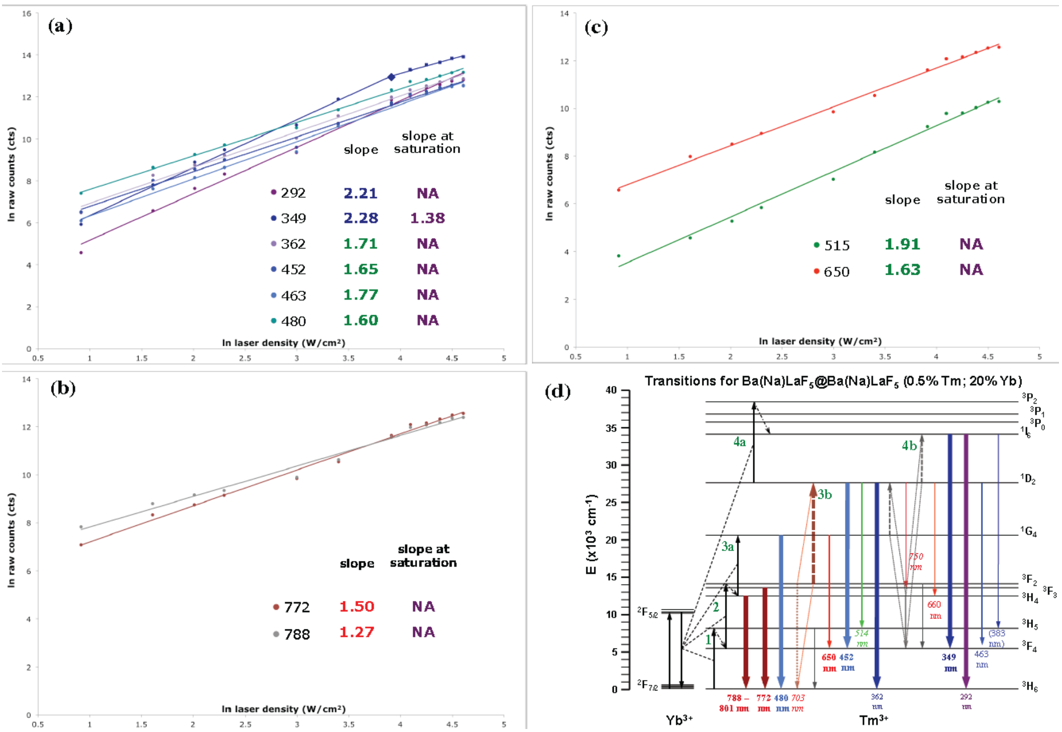

References

1. Hara, R.; Cady, G.H. Solubilities of salts in trifluoroacetic acid. *J. Am. Chem. Soc.* **1954**, *76*, 4285–4287.
2. Wang, Y.; Qin, W.P.; Di, W.-H.; Zhang, J.S.; Cao, C.-Y. Infrared-to-visible and infrared-to-violet upconversion fluorescence of rare earth doped LaF_3 nanocrystals. *Chin. Phys. B* **2008**, *17*, 3300–3305.
3. Vetrone, F.; Mahalingam, V.; Capobianco, J.A. Near-infrared-to-blue upconversion in colloidal $BaYF_5:Tm^{3+}$, Yb^{3+} nanocrystals. *Chem. Mater.* **2009**, *21*, 1847–1851.

© 2014 by the authors; licensee MDPI, Basel, Switzerland. This article is an open access article distributed under the terms and conditions of the Creative Commons Attribution license (http://creativecommons.org/licenses/by/3.0/).
